# Aggressive Invasive Pituitary Macroadenoma Involving Pituitary Infundibulum

**DOI:** 10.7759/cureus.69440

**Published:** 2024-09-15

**Authors:** Sanjay M Khaladkar, Sujith Kumar Samudrala

**Affiliations:** 1 Department of Radiology, Dr. D. Y. Patil Medical College, Hospital and Research Center, Dr. D. Y. Patil Vidyapeeth, Pimpri, Pune, IND

**Keywords:** aggressive pituitary tumor, pituitary gland, pituitary infundibulum, pituitary macroadenoma, pituitary tumor

## Abstract

Pituitary adenomas displaying radiographic invasiveness and an exceptionally high tumor growth rate are referred to as aggressive pituitary tumors (APTs). A pituitary adenoma involving the infundibulum is a rare occurrence. We report the case of a 41-year-old female presenting with headaches for one year, which were progressive in nature, along with blurring of vision. On radiological and histopathological examination, the cause was diagnosed as pituitary adenoma and APT involving the infundibulum.

## Introduction

The normal pituitary stalk tapers inferiorly. It appears wider at the level of the optic chiasma (3.25 + 0.56 mm) and tapers at insertion into the pituitary (1.91 + 0.40 mm). On T1-weighted images (T1WI), it is hypointense in relation to the optic chiasma and normal neurohypophysis. Because it does not have a blood-brain barrier, it exhibits significant homogeneous enhancement [1]. Pituitary stalk lesions can appear on an MRI as a uniform, V-shaped, circular, or pyramidal pattern. We discuss a case featuring a V-shaped enhancement of the infundibulum. Pituitary stalk lesions are rare entities. In a study conducted by Turcu et al., 92 pituitary stalk lesions were observed, of which nine were caused by macroadenoma [2].

## Case presentation

**Figure 1:**
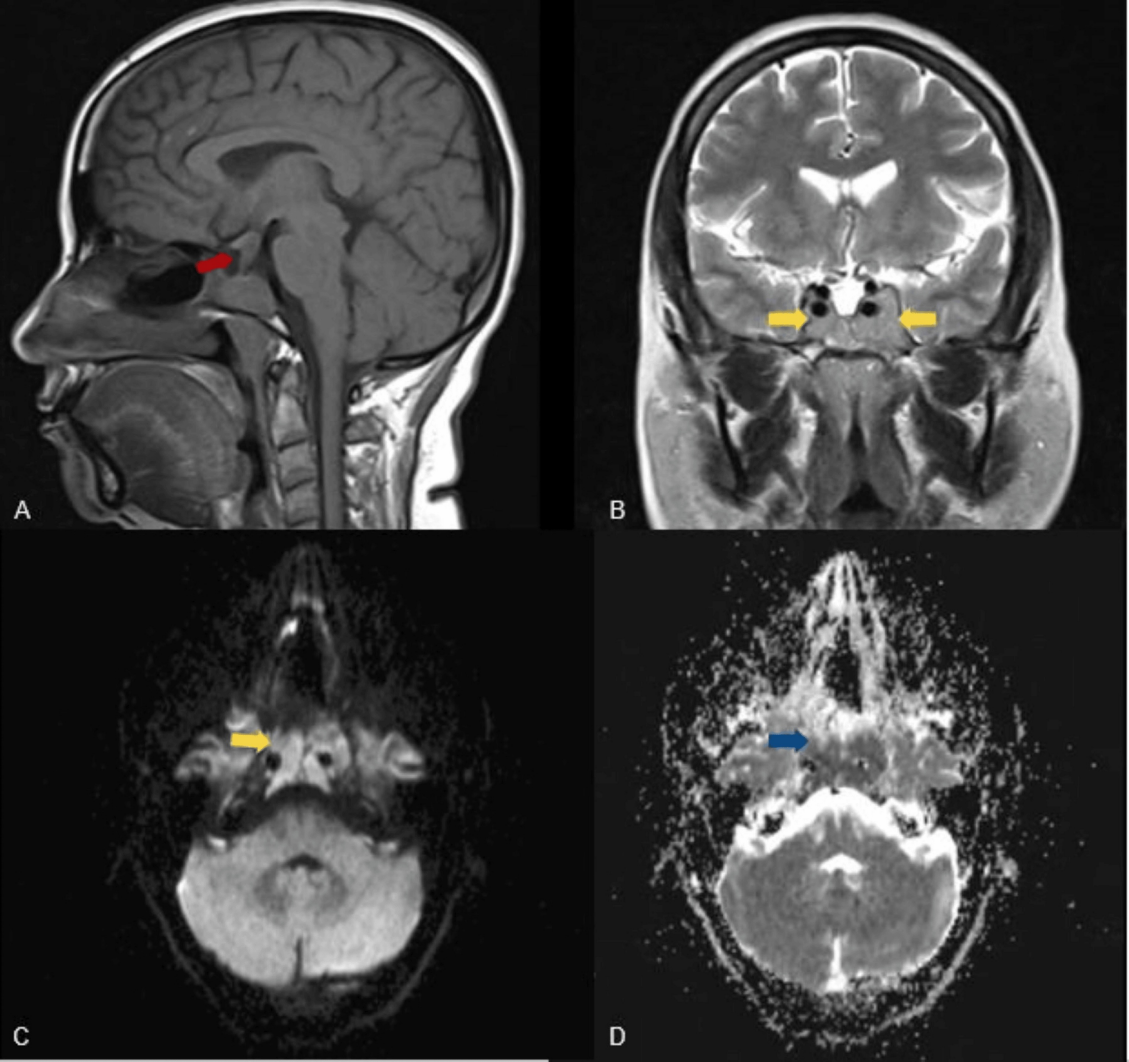
MRI findings - 1 Images showing pituitary macroadenoma involving infundibulum (red arrow) and bilateral cavernous sinuses (yellow arrows), showing diffusion restriction on DWI (green arrow) with low ADC values (blue arrow). A: sagittal T1; B: coronal T2; C: DWI; D: ADC ADC: apparent diffusion coefficient; DWI: diffusion-weighted imaging; MRI: magnetic resonance imaging

**Figure 2:**
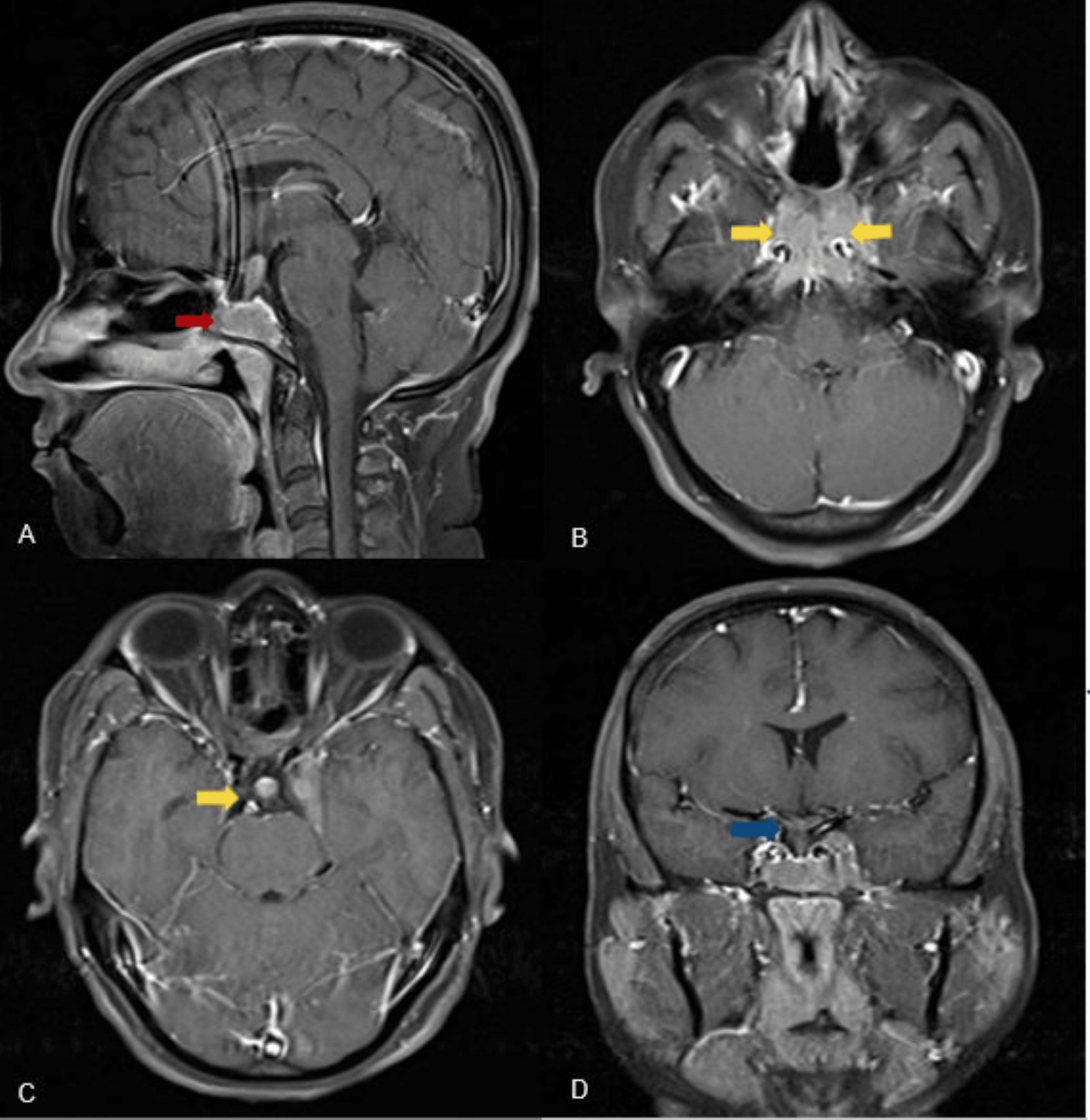
MRI findings - 2 Post-contrast T1 fat saturation sequences showing pituitary macroadenoma showing near-homogeneous post-contrast enhancement (red arrow), and thickening and enhancement of infundibulum (yellow arrows) with extension into bilateral cavernous sinuses (blue arrow). A: sagittal; B and C: axial; D: coronal MRI: magnetic resonance imaging

A 41-year-old female patient who had been asymptomatic until a year ago presented with progressively worsening headaches and blurring of vision. MRI of the brain with the pituitary was performed (Figures 1-2). Post-contrast dynamic imaging was performed in the coronal plane of the pituitary gland (Figure 2). A solid lesion measuring approximately 27 × 31 × 14 mm (anteroposterior × transverse × cranio-caudal, respectively) was noted in the pituitary fossa (Figures 1-2). The normal pituitary gland and posterior bright spot (hyperintensity on T1WI) of the pituitary were not visualized. It was isointense to hypointense on T1WI (Figure 1A) and hyperintense on T2WI (Figure 1B) and FLAIR with restricted diffusion on diffusion-weighted imaging showed low apparent diffusion coefficient values (Figures 1C-1D).

Contrast-enhanced MRI showed near-homogenous enhancement (Figure 2A). The adenoma extended bilaterally into the cavernous sinus (Figure 2D), encasing bilateral portions of the bilateral internal carotid artery (ICA) (left and right; Knosp grade IV). The lateral borders of the cavernous sinus showed an outward bulge bilaterally. Posteriorly, the adenoma involved the clivus. Inferiorly, it was eroding the pituitary floor and the dorsum sella, extending to the floor of the sphenoid sinus (defects were observed in the posteroinferior region of the nasal septum and the anterior wall of the sphenoid sinus after surgery). The infundibulum appeared thickened, measured approximately 7 × 8 mm (anteroposterior x transverse, respectively), and showed homogenous post-contrast enhancement (Figures 2B-2C). Optic chiasma and the intracranial portion of bilateral optic nerves appeared normal.

**Figure 3:**
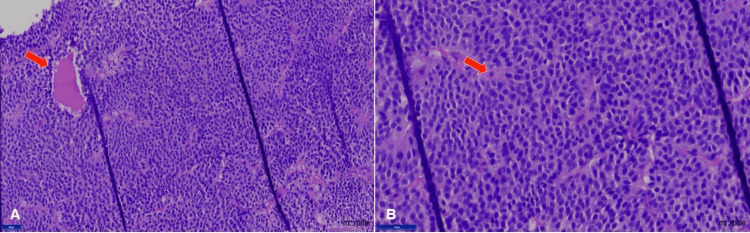
Histopathological examination of the biopsy with hematoxylin & eosin stain (H&E) (A) Original magnification x 40: photo micrograph showing tumor arranged in solid sheets and forming perivascular pseudorosettes. (B) Original magnification x 80: photo micrograph showing uniform polygonal tumor cells with round to oval vesicular nuclei and moderate amount of eosinophilic cytoplasm

Except for the issues reported above, the intracranial anatomy was normal. The diagnosis of an invasive pituitary macroadenoma was provided. The patient had previously undergone surgery for a pituitary macroadenoma five years earlier, the details of which were not available. A histopathology report done five years earlier showed foci of tumor cells arranged in solid sheets and forming perivascular pseudorosettes (Figure 3A). Individual tumor cells are uniform polygonal cells with round-to-oval vesicular, eccentrically located nuclei and a moderate amount of eosinophilic cytoplasm (Figure 3B). A tumor biopsy was suggestive of a pituitary adenoma.

## Discussion

Pituitary stalk involvement is observed in inflammatory, neoplastic, and infectious etiologies. The inflammatory causes include neurosarcoidosis, Wegener hypophysitis, xanthoma disseminatum, lymphocytic hypophysitis, Langerhans cell histiocytosis, and lupus cerebritis. Neoplastic causes include pituitary adenoma, craniopharyngioma, metastatic lymphoma, metastasis (from lung, breast, or unknown primary), germinoma, astrocytoma, neuronal neoplasm, and polyclonal hypergammaglobulinemia [3].

Aggressive pituitary tumors (APTs) are pituitary adenomas showing radiological invasiveness and an unusually rapid tumor growth rate. They account for 15% of all pituitary neoplasms. Pituitary adenomas are slow-growing, benign, and noninvasive. They occasionally exhibit aggressive behavior that includes invasion of nearby structures, resistance to standard care, and early and frequent recurrences. While exhibiting benign behavior, 25-55% of adenomas may display invasion of the dura, bone, and adjacent anatomical structures [4,5]. The features of APTs fall between those of benign pituitary adenomas and malignant pituitary carcinomas. Their clinical behavior is unique, exhibiting early postoperative recurrence, resistance to conventional treatment, and significant invasion of adjacent anatomical structures [5]. An MRI showing a pituitary adenoma with a Knosp grading of III-IV indicates that the tumor is invasive [6].

## Conclusions

We reported a rare case of aggressive invasive pituitary macroadenoma involving the pituitary infundibulum. To our knowledge, this is the first such case report from India. The key learning point from our case is that an aggressive invasive pituitary macroadenoma rarely involves the pituitary infundibulum. Clinicians should be aware of the various pathological conditions involving the infundibulum and the diverse presentations of aggressive invasive macroadenomas.
